# Efficacy of a Text Message-Delivered Extended Contact Intervention on Maintenance of Weight Loss, Physical Activity, and Dietary Behavior Change

**DOI:** 10.2196/mhealth.4114

**Published:** 2015-09-15

**Authors:** Lauren C Spark, Brianna S Fjeldsoe, Elizabeth G Eakin, Marina M Reeves

**Affiliations:** ^1^ Cancer Prevention Research Centre School of Public Health The University of Queensland Brisbane Australia

**Keywords:** weight, physical activity, diet, mobile telephone, intervention, behavior change, maintenance, SMS, mhealth, textmessaging

## Abstract

**Background:**

Extending contact with participants after the end of an initial intervention is associated with successful maintenance of weight loss and behavior change. However, cost-effective methods of extending intervention contact are needed.

**Objective:**

This study investigated whether extended contact via text message was efficacious in supporting long-term weight loss and physical activity and dietary behavior change in breast cancer survivors.

**Methods:**

Following the end of an initial 6-month randomized controlled trial of a telephone-delivered weight loss intervention versus usual care, eligible and consenting intervention participants received a 6-month extended contact intervention via tailored text messages targeting a range of factors proposed to influence the maintenance of behavior change. In this single-group, pre-post designed study, within group changes in weight, moderate-to-vigorous physical activity (Actigraph GT3X+ accelerometers), and total energy intake (2x24 hour dietary recalls) were evaluated from baseline to end of initial intervention (6 months), end of extended contact intervention (12 months), and after a no-contact follow-up (18 months) via linear mixed models. Feasibility of implementation was assessed through systematic tracking of text message delivery process outcomes, and participant satisfaction was assessed through semistructured interviews.

**Results:**

Participants at baseline (n=29) had a mean age of 54.9 years (SD 8.8), body mass index of 30.0 kg/m^2^ (SD 4.2), and were recruited a mean 16.6 months (SD 3.2) post diagnosis. From baseline to 18 months, participants showed statistically significantly lower mean weight (-4.2 kg [95% CI -6.0 to -2.4]; *P*<.001) and higher physical activity (mean 10.4 mins/day [95% CI 3.6-17.2]; *P*=.003), but no significant differences in energy intake (*P*=.200). Participants received a mean of 8 text messages every 2 weeks (range 2-11) and reported a high rate of satisfaction.

**Conclusions:**

In comparison to interventions without extended contact, results suggest text message–delivered extended contact may support the attenuation of weight regain and promote the maintenance of physical activity.

## Introduction

Maintaining a healthy body weight, engaging in regular physical activity, and eating a healthy diet are important for reducing the risk of chronic disease [1-3]. Behavioral lifestyle interventions are effective at promoting initial weight loss [4] and supporting physical activity (PA) and dietary behavior change [5,6]. However, maintaining improvements in these outcomes is often more difficult to achieve [7,8]. Regain in weight and relapses in health behaviors are common following the end of intervention. Trials indicate an average of 0.3 kg of weight is regained per month post intervention [9,10], and up to 50% of initial weight loss is regained within 1 year post intervention [11]. The challenge in maintaining weight loss has been largely attributed to the failure in maintaining PA and dietary improvements [12].

Extending contact with participants after an initial intervention has been found to improve weight loss maintenance [13,14] and support long-term PA and dietary behavior change [5,15]. A recent review of extended contact interventions delivered via telephone or face-to-face contact reported overall average weight regain was 3.2 kg less than in the corresponding control groups over approximately 18 months follow-up [13]. However, extended contact interventions delivered via face-to-face and telephone can be costly and time consuming [16,17], while Web-based delivery has been associated with poor participant retention and engagement [18,19]. Mobile phone text messaging may be an ideal extended contact intervention delivery modality due to its cost-effectiveness and ability to provide highly tailored support to participants in “real-time” [20,21]. Emerging evidence supports the feasibility and acceptability [22] and efficacy [23,24] of providing text message-delivered extended contact interventions to promote the maintenance of weight loss. However, no studies to date have explored the efficacy of a text message-delivered extended contact intervention to promote longer-term weight loss and associated PA and dietary behavior change.

This study aimed to assess the feasibility, acceptability, and efficacy of a 6-month text message-delivered extended contact intervention on the maintenance of weight loss and PA and dietary behavior change. This single-group, pre-post-designed study provided estimates of the effect sizes that may be achieved in a text message-delivered extended contact intervention and an opportunity to explore the relationship between text message dose and changes in weight and behavioral outcomes. The study aims were addressed in the context of the Living Well after Breast Cancer feasibility trial—a pilot randomized controlled trial evaluating a 6-month telephone-delivered weight loss intervention (versus usual care) for breast cancer survivors. Participants completing the 6-month telephone-delivered intervention were invited to receive a further 6-month intervention delivered via text messages. Long-term changes in weight, PA, and diet were evaluated within this intervention group. The maintenance of weight loss and associated behaviors is particularly important for breast cancer survivors as increasing evidence suggests that obesity, physical inactivity, and a poor diet quality are associated with increased risk of cancer recurrence and mortality [25-29].

## Methods

### Study Design

Participants in the Living Well after Breast Cancer feasibility trial completed a baseline assessment and were randomized to the initial telephone-delivered intervention (n=45) or usual care group (n=45). Those completing the initial telephone-delivered intervention (n=40) were invited to participate in the text message–delivered extended contact intervention. This sample size was insufficient to allow further randomization to an extended contact intervention versus control group. Assessments were conducted at baseline, 6 months (end of initial intervention), 12 months (end of extended contact intervention), and 18 months (end of no-contact follow-up). The Living Well after Breast Cancer feasibility trial and extended contact intervention were conducted at The University of Queensland in Brisbane, Australia. Ethical approval was obtained from the Human Research Ethics Committee of The University of Queensland and Queensland Health Research and Governance Unit.

### Participant Recruitment

The Living Well after Breast Cancer feasibility trial aimed to recruit overweight and obese women who had recently completed treatment for stages I-III breast cancer [30]. Women aged 18-75 years who had been diagnosed with stages I-III breast cancer in the previous 9-18 months and were living within a 50 km radius of the state capital, Brisbane, were identified from the Queensland Cancer Registry. Eligible women had a body mass index (BMI) of 25-40 kg/m^2^(ie, overweight or obese), had completed primary cancer treatment (ie, surgery, radiation, chemotherapy), and could speak sufficient English to participate in the intervention. Women were excluded if they had been diagnosed with ductal carcinoma in situ (stage 0) or with distant metastatic disease (stage IV), had a previous diagnosis of invasive breast cancer, had been diagnosed with any other cancer in the past 5 years, had contraindications to participating in unsupervised PA due to poor health or a medical condition, or self-reported a mental health condition that would interfere with their participation in the study. Women currently using or planning to use weight loss medications or those who had or were planning bariatric surgery were also excluded. To be eligible for the extended contact intervention, participants needed to own a mobile phone and be able to read a text message sent to that phone. Those eligible and agreeing to participate provided written, signed consent for the initial and extended contact intervention phases.

### Initial Weight Loss Intervention (Baseline to 6 Months)

The initial intervention aimed to promote weight loss through the combination of increased PA, reduced energy intake, and behavioral change strategies [14,31]. Intervention participants were mailed program materials (workbook, self-monitoring diary, scales, pedometer, calorie counter book) at the start of the intervention and received up to 16 telephone calls (6 x weekly calls followed by 10 x biweekly calls) from a coach using motivational interviewing techniques [32]. Coaches were Accredited Practicing Dietitians [30] who received additional training in motivational interviewing and exercise promotion. Participants were encouraged to aim for the targets of (1) weight loss of between 5-10% [33], (2) increasing moderate-vigorous PA to at least 30 minutes per day (210 minutes per week) [34], and (3) improving dietary behaviors (reducing energy intake by 2000 kJ [500 kcal] per day; <30% of total energy intake from fat; <7% of total energy from saturated fat; 5 servings of vegetables per day; 2 servings of fruit per day) [35,36]. Participants were provided with a target kilojoule intake (between 5000-7500 kJ/day [1200-1800 kcal/day]) based on their baseline weight and age [37].

### Extended Contact Intervention (6-12 Months)

The extended contact intervention was primarily delivered via individually tailored mobile phone text messages. The aim of this phase of the intervention was to promote sustained and/or ongoing improvements in weight loss and PA and dietary behavior change from the initial intervention. The design of the extended contact intervention was informed by literature on maintenance of weight loss and behavior change [38-40], a 2-week qualitative, formative study on text message usefulness and language with the target group (n=8), and Social Cognitive Theory [41].

At the start of the extended contact intervention, participants completed a tailoring telephone call with their coach to gather information to determine individual preferences for the content, timing, and frequency of text messages. The tailoring call and the resulting text messages focused on supporting participants to reach a longer-term (ie, 6 weeks) weight loss/weight maintenance goal and a short-term (ie, weekly) goal focused on either PA and/or dietary behaviors. To support participants to reach these goals, the text messages targeted self-regulation skills through nine evidence-based strategies [36,42-44]: prompt SMART (Specific, Measurable, Attainable, Realistic, Timely) goal setting, prompt self-assessment of goal attainment, provide feedback, prompt self-reward, prompt self-monitoring, prompt relapse prevention, prompt “real-time” planned behavior, prompt preparatory and planning behavior, and prompt barrier identification and solutions. Each of these strategies were discussed with the coach during the tailoring call, and individual’s responses (eg, self-nominated reward for reaching goal) were recorded and used to tailor the content of the text messages. These behavior change strategies were targeted in five different types of text messages (Table 1).

Message dose (ie, frequency) and timing (ie, day of week and time of day) were tailored to each participant for each of the five message types (Table 1). All participants received a minimum dose of 21 text messages over the 6-month intervention, including 12 weight self-monitoring, three goal checks for weight, three goal resets for weight and, depending on whether participants chose to focus on PA and/or dietary behaviors, they also received three goal resets for PA and/or three for diet (see examples in Table 1). In addition to this minimum dose, participants could choose to receive additional messages targeting PA and/or dietary behaviors, including goal checks (maximum for each behavior n=24) and cues for planned behaviors (maximum for each behavior n=48; Table 1).

The frequency of weight self-monitoring messages was based on research that supports regular self-monitoring of weight for long-term weight loss [38]. The frequency of texts for goal check for weight and goal reset for weight and behavior were at six weekly intervals to regularly encourage participants to reflect on the suitability of their SMART goals based on recent changes. Based on our formative study, the frequency of the goal checks for behavior change and cues for planned behaviors were to make regular, but not overwhelming, contact.

Participants were encouraged to reply to goal check messages for weight, physical activity, and/or diet (Table 1). If participants responded to these messages, a tailored goal check reply message was sent (Table 1). When participants replied to a goal check message in the requested format (ie, yes/no), the software sent an automatically tailored response. If participants replied to a goal check message in an unexpected format, the software was unable to send an automatic reply. In this case, an email was sent to the research team to manually trigger the correct reply or, where necessary, individual messages could be written and sent directly to participants if the triggered response was not appropriate. For example, if a participant reported being ill, the reply would mirror the preset content but was reworded to acknowledge the illness and send well wishes. This was done to ensure that the close rapport developed between participants and their coaches was maintained to ensure the accountability and “humanness” of the program was upheld (an important factor identified in formative research).

Participants were also encouraged to reply to the goal reset messages (Table 1), and these data were used to update the tailoring information about their goals, but they did not receive a tailored response. Individually tailored message content and sending schedules were entered into a Web-based software program specifically built for this study that interfaced with a commercial telecommunication gateway through MessageMedia Pty Ltd to allow the sending and receiving of messages to individual participants. To ensure message content remained relevant, participant tailoring information was updated during a 12-week tailoring telephone call with their coach.

**Table 1:** Examples of how self-regulation strategies were targeted across the five different types of text messages.

Text message type	Strategies targeted in this type of message	Example	Minimum dose over 6 months			Frequency		
			Weight	PA	Diet	Weight	PA	Diet
Self-monitoring of weight	Prompt self-monitoring	If u havent weighed yourself in the last 2 weeks Karen then do it today! Amy	12	N/A	N/A	Biweekly	N/A	N/A
Goal check	Prompt self-assessment of goal attainment	How r u going Karen? Reach ur goal 2 walk 3x30mins? Text me back yes or no. Amy	3	0	0	Once in weeks 6, 18, 24	Maximum 1 per week (participant determined)	Maximum 1 per week (participant determined)
Goal check reply	Provide feedback	Yes example: Fantastic Karen! Regular exercise will help u control ur weight. Remember 2 buy a magazine & reward yourself 4 ur excellent effort. Amy No example: Don’t worry Karen. Think about what got in the way & plan 4 it this week. Work around ur barriers 2 achieve ur goals. Amy	Triggered by participant’s reply			Triggered by participant’s reply		
	Prompt self-reward							
	Prompt relapse prevention							
	Prompt “real-time” planned behavior							
	Prompt self-monitoring							
	Prompt preparatory and planning behavior							
	Prompt barrier identification and solutions							
Goal reset	Prompt SMART goal setting	Reflect on ur exercise goals Karen. Eventually u want 2 aim for 7x30mins exercise/week & more is better. Text me back with a new goal! Amy	3	3	3	Once in weeks 6, 18, 24	Once in weeks 6, 18, 24	Once in weeks 6, 18, 24
Cues for planned behavior	Prompt “real-time” planned behavior	Want 2 feel more energised Karen? Make time 2 walk 3x30mins this week & feel the difference. Amy	N/A	0	0	N/A	Maximum 2 per week (participant determined)	Maximum 2 per week (participant determined)
	Prompt self-monitoring							
	Prompt preparatory and planning behavior							
	Prompt barrier identification and solutions							

### Data Collection and Outcomes

#### Feasibility Measures

Feasibility of implementation was assessed in relation to uptake of the intervention (ie, consent rate and characteristics of those who consented to the extended contact intervention), and process outcomes related to the delivery of the extended care intervention (ie, the number of messages sent, the rate of replies to goal check and goal reset messages, the rate of researcher intervention required to trigger goal check replies or alter content of text messages, and the duration of the initial and check-in telephone calls).

#### Efficacy Measures

##### Overview

Data were collected at baseline, 6, 12, and 18 months by trained research staff. Data collection involved an in-person assessment, two telephone interviews, and wearing an activity monitor for a period of 7 days. Intervention participants received printed, tailored feedback on weight and behavioral outcomes following all assessments.

##### Weight

Weight was measured in duplicate, without shoes or heavy clothing, using standard calibrated scales (nearest 0.1 kg).

##### Physical Activity

Physical activity was measured using a tri-axial accelerometer (GT3X+, Actigraph), worn for 7 consecutive days during waking hours. Data were used to determine minutes per day spent in moderate-vigorous PA (counts ≥1952) [45,46]. Average moderate-vigorous PA on valid days (ie, 10+ hours of wear) was then multiplied by 7 to yield a weekly estimate.

##### Energy Intake

Energy intake was measured using two, unprompted 24-hour dietary recalls (recalling 1 weekday and 1 weekend day). The dietary recalls were conducted via telephone using FoodWorks Interview (version 1, 2009, Xyris Software), based on a 5-stage multipass method [47]. Participants were provided with a food model booklet to assist in portion size estimation and food quantities. Dietary intake was analyzed using Foodworks Professional Edition (version 6, 2009, Xyris) nutritional analysis software to determine total daily energy intake. The average of energy intake over the 2 recalled days was used.

#### Participant Acceptability Measures

At the 12-month assessment, participants were invited to participate in a one-on-one semistructured interview to assess satisfaction with the extended contact intervention. A 5-point Likert scale was also used to assess the helpfulness of the text messages (from 1 “very unhelpful” to 5 “very helpful”).

#### Statistical Analyses

The sample size for the extended contact intervention was determined by the number of participants completing the initial intervention (n=40). Data analysis was performed using SPSS for Windows (version 21), and statistical significance was set at *P*<.05 (two-tailed). Changes from baseline at 6, 12, and 18 months for each outcome are reported. Change scores (follow-up minus baseline) had approximately normal distributions. Changes from 6 to 12 months and from 12 to 18 months are also reported. Data were analyzed, separately for each outcome, using linear mixed models, with random intercepts for each subject to account for repeated measures. Models included time (baseline, 6, 12, or 18 months) and adjusted for baseline values, age, income, time since diagnosis, and chemotherapy treatment. These latter variables were included to correct for observed changes in group composition between 6, 12, and 18 months caused by dropout [48]. This method was used to handle missing data rather than baseline-value-carried-forward, which can overstate maintenance by not allowing participants who drop out to experience any decline (or gain). The association of dose of number of text messages received (treated as a continuous variable) with change in weight and PA and diet from baseline was examined by adding this variable to the linear mixed models. Process outcomes were evaluated descriptively. Participant acceptability was determined through semistructured interview questions, and thematic analysis of the qualitative interview data followed a systematic and iterative process [49]. This technique involved identifying major themes and categories from each participant and then examining common and uncommon themes across the complete dataset.

## Results

### Feasibility Outcomes

#### Participant Recruitment and Characteristics

**Figure 1:**
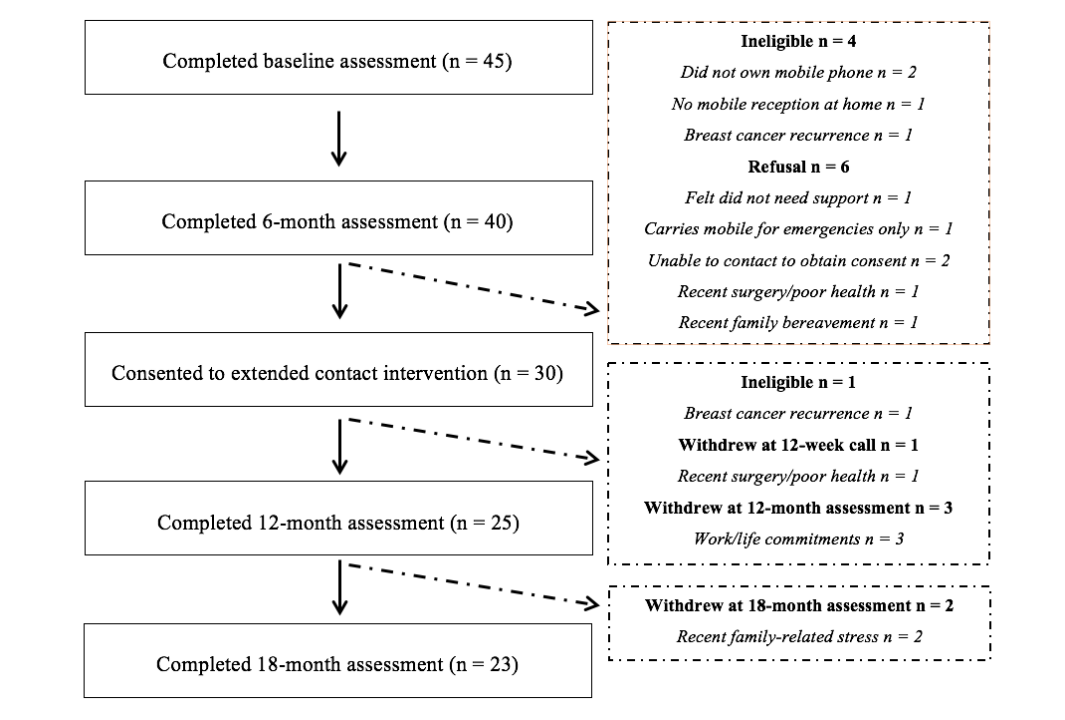
Flow of participants from baseline to the final 18-month assessment.

Figure 1 shows the flow of participants through the study. A total of 45 women were randomized to the initial intervention, with 40 (88%) completing the 6-month assessment. Of these women, 36 were eligible to participate in the extended contact phase and 30 (83%) consented to participate, with one participant later becoming ineligible due to a recurrence. Twenty-five (86%) extended contact intervention participants completed the 12-month assessment, and 23 (79%) completed the 18-month assessment.

Participants at baseline had a mean age of 54.9 years (SD 8.8), a BMI of 30.0 kg/m^2^(SD 4.2), and were recruited a mean 16.6 months (SD 3.2) post diagnosis and 7.1 (1.4) months post treatment completion (Table 2 [50]). Compared to participants who completed all follow-up assessments (n=23), participants who withdrew (n=6) were more likely to be younger (mean 56 years vs 49 years), have a lower BMI (mean 30.5 kg/m^2^vs 28.2 kg/m^2^), be employed (65% vs 100%), and were less likely to have a high household income (32% vs 17%).

**Table 2:** Baseline characteristics of participants who consented to the extended contact intervention (n=29).

Characteristics		Mean (SD) or % (n)
Age in years		54.9 (8.8)
Weight in kg		81.8 (13.1)
BMI, kg/m^2^		30.0 (4.2)
**Time in months**		
	Since diagnosis	16.6 (3.2)
	Since treatment	7.1 (1.4)
Caucasian		97% (28)
Married/living together		83% (24)
High household income (>AU$2391+/wk)^a^		28% (7)
**Education**		
	Completed high school	76% (22)
	Completed tertiary education	34% (10)
Employed (full-time, part-time, casual)		72% (21)
Post-menopausal		59% (17)
**Stage**		
	Stage I	48% (13)
	Stage II-III	52% (14)
**Treatment**		
	Mastectomy	38% (11)
	Chemotherapy	62% (18)
	Radiation	79% (23)
	Endocrine therapy	69% (20)

^a^Household income of >AU$2391+ / week is within the top two quintiles based on the Australian population [50].

#### Extended Contact Intervention Process Outcomes

Participants chose to receive text messages focused on both PA and diet (n=12), or focused only on PA (n=11) or diet (n=6). Completing participants (n=25) received an average of 74 (range 25-135) text messages over the 6-month intervention. This equated to an average of approximately 8 text messages every 2 weeks (range 2-11), typically consisted of one weight self-monitoring, three cues for planned behavior, two goal checks, one tailored goal check reply, and one goal reset. Overall, participants replied to every two in three goal check messages (67% response rate), and every one in five goal reset messages (20% response rate). Less than half of the overall goal check reply messages were automatically sent to participants (41%), with researcher intervention required most of the time to either trigger the appropriate “yes” or “no” goal check reply (36%) or alter the content of the message to be appropriate to the participant reply (23%).

The initial telephone consultation with the coach lasted 22 minutes on average (minimum-maximum: 11-40 minutes). The 12-week check-in telephone call lasted 16 minutes on average (minimum-maximum: 8-31 minutes), and 88% of participants (n=22) used this call to update their goals or alter their text message content. The requested changes during this call were mostly to change the behavioral focus of messages (eg, from PA- to diet-focused; n=3), or to increase (n=2) or decrease (n=2) the frequency of messages received. Two participants were not able to be contacted to receive a 12-week check-in telephone call, and their tailoring information remained the same for the full 6 months.

### Efficacy Outcomes

#### Weight

**Figure 2:**
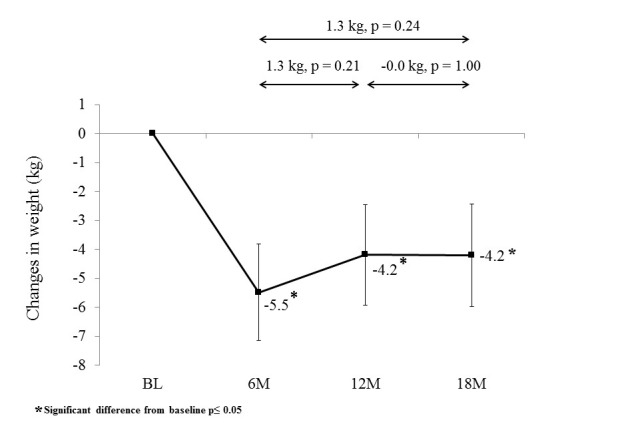
Change in weight (kg) from baseline (BL) to 6 months (6M), 12 months (12M), and 18 months (18M) follow-up.

Overall, mean weight at 6, 12, and 18 months was statistically significantly lower than baseline (Figure 2; Multimedia Appendix 1). There was a small but non-significant increase in weight during the extended contact intervention (1.3 kg [95% CI -0.5 to 3.1]; *P*=.211), with weight remaining relatively stable over the no-contact follow-up period (-0.1 kg [95% CI -1.9 to 1.8]; *P*=1.000). Participants lost a mean of 6.8% (95% CI 4.7-8.8) of baseline weight during the initial intervention and regained 1.6% (95% CI -0.6-3.8) of baseline weight (23.5% of initial weight lost) during the extended contact intervention. At 18 months, participants had statistically significantly lower mean weight (-4.2 kg [95% CI -6.0 to -2.4]; *P*<.001) compared to baseline and on average had lost 5.2% (95% CI 3.0-7.4) of body weight.

#### Physical Activity

**Figure 3:**
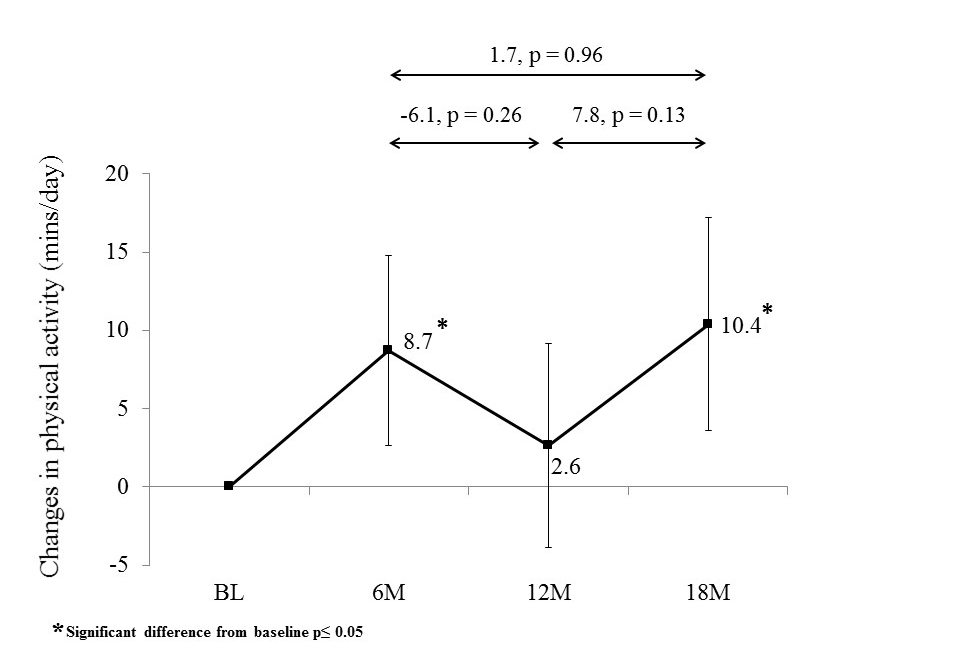
Change in physical activity (mins/day) from baseline (BL) to 6 months (6M), 12 months (12M), and 18 months (18M) follow-up.

Participants significantly increased their moderate-to-vigorous PA from baseline at 6 and 18 months (Figure 3; Multimedia Appendix 1). Physical activity at 12 months was not significantly different to baseline. Physical activity decreased but not significantly during the extended contact intervention (-6.1 mins/day [95% CI -14.9 to 2.8]; *P*=.260) and increased but not significantly during the no-contact follow-up (7.8 mins/day [95% CI -1.6 to 17.2]; *P*=.132).

#### Diet

**Figure 4:**
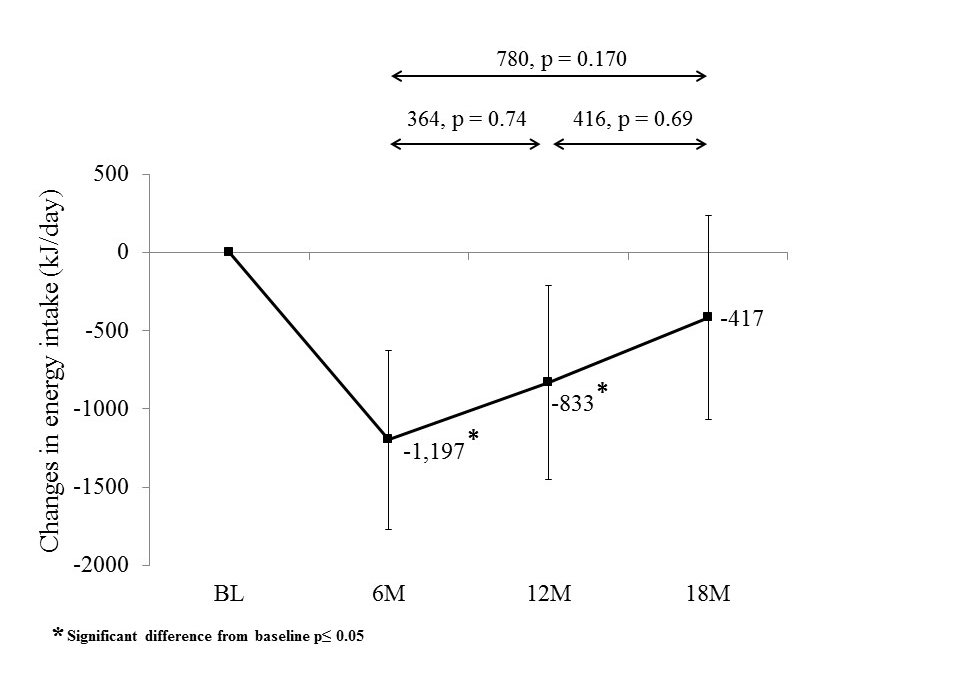
Change in energy intake (kJ/day) from baseline (BL) to 6 months (6M), 12 months (12M), and 18 months (18M) follow-up.

Participants significantly decreased their energy intake from baseline at 6 months and 12 months (Figure 4; Multimedia Appendix 1). Energy intake at 18 months was not significantly different to baseline. Energy intake increased, but not statistically significantly, during both the extended contact intervention (364 kJ/day [95% CI -609 to 1338]; 87 kcal/day [95% CI -146 to 320]; *P*=.735) and the no-contact follow-up (416 kJ/day [95% CI -620 to 1451]; 99 kcal/day [95% CI -148 to 347]; *P*=.690).

#### Text Message Dose

Each additional text message received per week was associated with 9.5 minutes per day (95% CI 3.1-15.8; *P*=.004] more PA at 18 months. There was no significant association between weekly text message dose and change in weight (1.3 kg [95% CI -0.3 to 2.8]; *P*=.098) or change in energy intake (279 kJ/day [95% CI -157 to 716]; 67 kcal/day [95% CI -37 to 171]; *P*=.198).

### Participant Acceptability Outcomes

Of the 25 participants completing the extended contact intervention, 80% reported the extended contact as either “very helpful” (6/25, 24%) or “helpful” (14/25, 56%), and 20% (5/25) reported the extended contact as “neither helpful nor unhelpful”. The majority of women reported in the semistructured interviews that the text messages primarily served as a prompt or reminder for a specific behavioral cue to action, fostered accountability to keep on track, and that the text message content was sufficiently personalized. Overall, participants highly valued the goal check reply messages and the 12-week check-in telephone call as they provided a “human” element of contact important for ongoing feedback and accountability.

## Discussion

### Principal Considerations

The aim of this study was to explore the feasibility, acceptability, and efficacy of a 6-month text message–delivered extended contact intervention in promoting the maintenance of weight loss and PA and dietary behavior change in breast cancer survivors who completed an initial 6-month telephone-delivered weight loss intervention. Overall, results suggest extended contact may have helped to attenuate weight regain and promote the maintenance of long-term change in PA. The highly tailored text message–delivered extended contact intervention was also feasible to deliver and acceptable among this sample of primarily older breast cancer survivors.

Importantly, mean weight at 18-months follow-up was significantly lower than at baseline (approximately 5.2% of initial body weight loss). Evidence from a large number of previous weight loss trials suggests that average weight regain following an intervention is in the order of 0.3 kg per month post intervention [9,10] or approximately 50% of weight lost is regained within 12 months post intervention [51]. In comparison, the magnitude of weight regain observed here was considerably less (approximately 0.1 kg per month or 23.5% regain of initial weight loss over a 12-month period). This study adds to the limited evidence to date on the efficacy of text messaging to support weight loss maintenance.

Donaldson et al [23] examined a 3-month text message-delivered extended contact weight loss intervention following an initial 3-month face-to-face weight loss intervention, finding that it resulted in an additional 1.6 kg weight loss at the end of the extended contact intervention compared to a regain of 0.7 kg weight regain in the no-contact control group [23]. A 1-month text message-delivered behavior change intervention following a commercially available weight loss program reported 87% of participants regained less than 3% of initial weight loss at 3-months follow-up [24]. However, these studies focused on relatively short-term maintenance outcomes making comparison with outcomes here difficult [23,24]. Together, these findings provide emerging support for the use of text messaging to deliver extended contact interventions to promote weight loss maintenance.

Reporting on changes in PA and energy intake (the behaviors that underpin weight loss maintenance) following extended contact interventions has been limited. Overall, studies suggest that changes in PA are largely maintained at the end of an extended contact intervention [9,52-57], but maintenance of dietary behavior change appears more challenging [9,56,57]. Contrary to these previous findings, PA in this study had relapsed by the end of extended contact but rebounded by follow-up, while the opposite pattern of behavior was observed for energy intake. This inconsistent pattern of PA and dietary behavior change to weight change is mirrored in findings from the broader weight loss maintenance literature [9,56,57]. However, it is important to acknowledge the long-standing caveat of correlating changes in PA and diet as measured at a single point in time with more cumulative changes in weight.

Determining significant dose-response relationships between text message dose and behavioral outcomes may help inform the development of future extended contact interventions. Every additional text message received per week in this study was associated with a mean increase of more than 1 hour of PA per week at follow-up. This suggests the dose of extended intervention contact received may influence the maintenance of long-term behavior change outcomes but has yet to be examined in other studies. A larger scale intervention that allows personalized tailoring could examine the minimum dose of text messages received whereby a significant maintenance effect is no longer observed.

The acceptability of the delivery modality to the target group also influences intervention success [7]. Consistent with findings from previous studies exploring text message-delivered extended contact interventions to promote the maintenance of weight loss and behavior change [22,23], satisfaction ratings were high. Notably, the intervention completion rate was higher than previously reported in a younger population (86% vs 58%; [23]). This is promising given skepticism regarding the suitability of text message-delivered interventions in older adults [58].

Participants received a wide range of text message doses, and this reflects the feasibility to deliver a highly tailored extended contact intervention. Participant engagement with replying to messages was high, although researcher intervention was often required to provide suitably tailored feedback. Future text message-delivered extended contact interventions should integrate more sophisticated software and programming approaches, such as those applied in mobile phone app behavior change tools to improve automation while maintaining a high level of participant tailoring [59,60]. However, our qualitative findings suggest that technology-driven interventions should maintain an element of “human” connection to foster ongoing participant satisfaction and accountability. A highly automated text message-delivered extended contact intervention may therefore need to be supplemented with additional non-automated contact, such as that delivered via telephone or email.

### Limitations

A number of study limitations should be considered in interpreting the results. This study was largely exploratory as it was not feasible to re-randomize participants following the initial intervention due to the small sample size in the larger trial in which this study was embedded. Thus, comparison to a true control group was not possible. Establishing intervention acceptability among an older population group such as breast cancer survivors is a strength of the study but may also limit the generalizability of results to broader populations. Measurement error may have contributed to differences in patterns and magnitude of behavioral outcomes. Comparisons of behavior change outcomes to previous literature were difficult due to the limited number of studies that report post-intervention behavioral outcomes. This highlights the importance of future studies including post-intervention assessments to further examine how patterns of behavior change may influence weight loss maintenance.

### Conclusions

In summary, findings from this study support the feasibility, acceptability, and provide preliminary evidence on efficacy of a text message-delivered extended contact intervention to promote the maintenance of weight loss and PA among a predominately older female subgroup. There is a growing evidence base supporting the utility of text messaging as an intervention delivery modality [20,21,61,62], with this study being the first to report on outcomes of a text message-delivered extended contact intervention to support long-term maintenance of weight loss and behavior change. Results suggest providing extended contact via text messaging after an initial intensive weight loss intervention may help attenuate weight regain and promote long-term physical activity behavior change compared to what otherwise would be observed without extended contact. Randomized controlled trials with larger and more diverse samples are needed, along with comparative effectiveness and cost-effectiveness trials comparing text messaging with other delivery modalities that might be suited to extended contact interventions (eg, mobile phone apps).

## Appendix

Multimedia Appendix 1 Mean changes in weight, physical activity, and diet from baseline (BL) to 6 months (6M), 12 months (12M), and 18 months (18M) follow-up.

## Abbreviations

BMI: body mass index
PA: physical activity
SMART: Specific, Measurable, Attainable, Realistic, Timely
