# Supplementary material for: Efficacy of a Text Message-Delivered Extended Contact Intervention on Maintenance of Weight Loss, Physical Activity, and Dietary Behavior Change
Source: JMIR Mhealth Uhealth. 2015 Sep 15;3(3):e88. doi: 10.2196/mhealth.4114 (PMC4705007; doi:10.2196/mhealth.4114)
Supplement: Multimedia Appendix 1 [file mhealth_v3i3e88_app1.pdf]

Multimedia Appendix 1. Mean changes in weight, physical activity, and diet from baseline (BL) to 6-months (6M), 12-months (12M) and 18-months (18M) follow-up.

		**Weight (kg)**	**MVPA (mins/day)**	**Energy intake (kJ/day)**
Baseline	**Mean ±SD**	81.7 ± 13.1	27.3 ± 18.8	7862 ± 1982
6MΔ [a]	**Mean (95% CI)**	-5.5 (-7.2, -3.8)	8.7 (2.6, 14.8)	-1197 (-1768, -626)
	**p**	<0.001	0.006	<0.001
12 MΔ [a]	**Mean (95% CI)**	-4.2 (-5.9, -2.5)	2.6 (-3.9, 9.2)	-833 (-1452, -214)
	**p**	<0.001	0.423	0.009
18 MΔ [a]	**Mean (95% CI)**	-4.2 (-6.0, -2.4)	10.4 (3.6, 17.2)	-417 (-1067, 233)
	**p**	<0.001	0.003	0.200
12MΔ-6MΔ [b]	**Mean (95% CI)**	1.3 (-0.5, 3.1)	-6.1 (-14.9, 2.8)	364 (-610, 1338)
	**p**	0.211	0.260	0.735
18MΔ-12MΔ [b]	**Mean (95% CI)**	-0.0 (-1.9, 1.8)	7.8 (-1.6, 17.2)	416 (-620, 1452)
	**p**	1.000	0.132	0.690
18MΔ-6MΔ [b]	**Mean (95% CI)**	1.3 (-0.5, 3.1)	1.7 (-7.5, 10.9)	780 (-223, 1784)
	**p**	0.242	0.957	0.170

Footnote: Analyses carried out under intention-to-treat principles for all participants at baseline and 6-months, and then completers from this sample at 12- and 18-months [BL=29; 6M=29; 12M=25; 18M =23]

[a] Mean changes from baseline, estimated from mixed models, adjusted for baseline values, age, income, time since diagnosis, and chemotherapy treatment [b] Differences in change between follow-up time points, estimated from mixed models, adjusted for baseline values, age, income, time since diagnosis, and chemotherapy treatment
